# Two-Photon Polymerization of Albumin Hydrogel Nanowires Strengthened with Graphene Oxide

**DOI:** 10.3390/biomimetics6040066

**Published:** 2021-11-24

**Authors:** Nikita Nekrasov, Natalya Yakunina, Vladimir Nevolin, Ivan Bobrinetskiy, Pavel Vasilevsky, Alexander Yu. Gerasimenko

**Affiliations:** 1 Center for Probe Microscopy and Nanotechnology, National Research University of Electronic Technology, 124498 Moscow, Russia; 8141147@gmail.com (N.N.); natali.swan.1999@mail.ru (N.Y.); vkn@miee.ru (V.N.); bobrinet@biosense.rs (I.B.); 2 BioSense Institute-Research and Development Institute for Information Technologies in Biosystems, University of Novi Sad, 21000 Novi Sad, Serbia; 3 Institute of Biomedical Systems, National Research University of Electronic Technology, 124498 Moscow, Russia; pavelvasilevs@yandex.ru; 4 Institute for Bionic Technologies and Engineering, I.M. Sechenov First Moscow State Medical University, Bolshaya Pirogovskaya Street 2-4, 119991 Moscow, Russia

**Keywords:** two-photon polymerization, bovine serum albumin, graphene oxide, filament propagation, nanowires, mechanical properties, electrical properties

## Abstract

Multifunctional biomaterials can pave a way to novel types of micro- and nanoelectromechanical systems providing benefits in mimicking of biological functions in implantable, wearable structures. The production of biocomposites that hold both superior electrical and mechanical properties is still a challenging task. In this study, we aim to fabricate 3D printed hydrogel from a biocomposite of bovine serum albumin with graphene oxide (BSA@GO) using femtosecond laser processing. We have developed the method for functional BSA@GO composite nanostructuring based on both two-photon polymerization of nanofilaments and direct laser writing. The atomic-force microscopy was used to probe local electrical and mechanical properties of hydrogel BSA@GO nanowires. The improved local mechanical properties demonstrate synergistic effect in interaction of femtosecond laser pulses and novel composite structure.

## 1. Introduction

Bio-inspired nanoelectromechanical devices (NEMS) are actively rising technology in advanced functional systems paving the way for modern technology of soft, stretchable, self-healing next-generation systems [1,2]. Using nanocarbon materials like carbon nanotubes (CNT) and graphene in complex composites improves its electro-active and scaffold properties. Recently these materials were successfully implemented in the development of pH sensitive hydrogels where swelling rate is dependent either on alkaline or acid environments [3,4].

Albumin is a protein that finds its application in different areas of bionanohybrids production and state-of-the-art nanotechnology [5]. Albumin is widely applied to laser-welded biological tissues as a laser solder [6]. Polymerization of bovine serum albumin (BSA) molecules is possible either by direct single-photon processing under UV irradiation [7], thermal treatment [8], or two-photon polymerization (TPP) [9,10]. The later process requires a photoinitiator (PI) [11]. Two-photon polymerization of BSA is actively employed to develop devices, such as microactuators [12] and optical nanodevices [9,13]. BSA-based microdevices have demonstrated a high environmental pH sensitivity, that makes BSA composites ideal materials for stimuli-sensitive biocompatible applications [9]. Femtosecond laser programmed artificial musculoskeletal system was proposed for soft smart muscle based on pH-responsive BSA [10].

Previously we demonstrated that CNT/BSA composites with varied conductivity and concentration can be used as biocompatible materials for sensor application [14] or scaffold for cells growth [15]. The 2D materials, such as graphene, provide advanced properties of composites for lower load concentration while maintaining flexibility and stretchable properties. Different methods were suggested for development of graphene/albumin aggregates, either by hydrophobic interactions [16], Van der Waals [17], or chemical crosslinking [18,19]. The graphene in form of graphene oxide (GO) is actively used in composites formation due to its hydrophilic properties, while for conductive properties the subsequent reduction is needed. The reduction of GO by standard chemical methods was suggested in the process of mixing with hydrogel materials [3]. In addition, the effect of ionic strength on electrostatic interaction of albumin with GO results in albumin binding energy modulation and increase of protein absorption density for higher ionic strength of solutions [20]. Nevertheless, the development of nanostructured functional composites based on proteins and graphene is still a challenging task.

In this work, we suggest the use of femtosecond pulsed laser for hydrogel nanowires formation using two-photon polymerization of albumin and graphene oxide composites. The local electrical and mechanical properties of BSA and hydrogel nanowires were investigated. The improvement of mechanical properties for BSA@GO hydrogel nanowires was demonstrated even at low GO concentration. 

## 2. Materials and Methods

BSA powder was purchased from BioClot (Aidenbach, Germany). GO was provided by Smagulova S.A. (M.K. Ammosov North-Eastern Federal University, Yakutsk, Russia) in 4.7 g/L water solution. Photoinitiator (methylene blue (MB), 1% water solution) was purchased from LLC Zoomir (Moscow, Russia).

**Figure 1:**
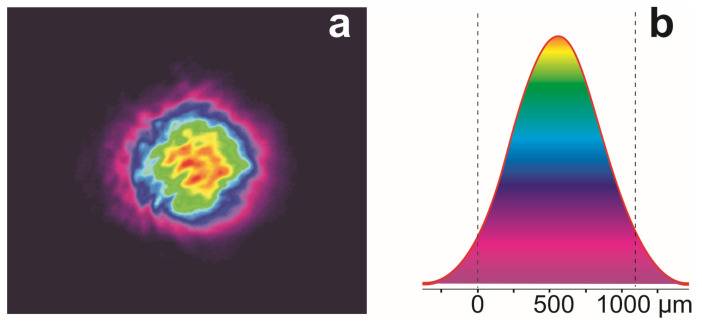
2D beam profile visualization (**a**) and Gaussian shape of the initial beam (**b**) of femtosecond laser.

Two-photon polymerization was performed on tunable Ti:Sapphire femtosecond laser Chameleon (Coherent, Santa Clara, CA, USA) with wavelength set to 715 nm with 140 fs pulse duration, 80 MHz repetition rate, and varied power up to 30 mW. To tune the power of the laser radiation, we used a motorized optical attenuator OAGP-M based on Glan prism (Avesta, Moscow, Russia). The initial beam had a Gaussian profile (Figure 1). The beam diameter at the 1/e^2^ level was 1.2 ± 0.1 mm. The images of the spatial profile of the laser beam were obtained using an SP620U CCD camera (Ophir Optronics Solutions, Jerusalem, Israel). The pulse repetition rate was controlled using an MSO6054A oscilloscope (Agilent Technologies, Santa Clara, CA, USA). Samples were placed on the motorized XY 8MTF scanning stage (Standa, Vilnius, Lithuania). The laser pulses reached the sample surface through an optical upright microscope with 60× objective (NA = 0.65). The accumulated energy for TPP at average pulse power varied via the scanning speed of XY stage. A SVETOLIT-50 mercury lamp (Svetolit, Moscow, Russia) was used for UV experiments.

BSA was dissolved in 1× PBS (phosphate buffer saline) (pH = 7.4) in the concentration of 0.6 g/mL. To avoid the fast aggregation of proteins, we added 100 mg of BSA in 1 mL of PBS followed by ultrasonic treatment for 5 min after each step. MB was added to BSA solution to reach the 16 mM concentration of MB. One hour of ultrasonic treatment in cold water was used to prevent the denaturation of proteins. To produce BSA@GO solution, 10 µL of GO solution was added to 1 mL of PBS prior to BSA to reach 0.005% concentration of graphene.

BSA hydrogel nanowires were characterized via atomic force microscopy (AFM, Solver Pro (NT-MDT, Moscow, Russia)). Two types of silicon cantilevers were used: standard silicon cantilevers with resonance frequency F_res_ = 200 kHz (NSG01, TipsNano, Tallin, Estonia) for force-distance curves and with 35 nm Au coating and F_res_ = 120 kHz (NSG03/Au) for conductive measurements. Raman spectra was measured on microRaman spectrometer Centaur HR (Nanoscan Technology, Dolgoprudny, Russia) with a 100× objective at 532 nm (Cobolt, Solna, Sweden) with a beam spot of ∼1 μm^2^ and laser power of 0.5 mW.

## 3. Results and Discussion

**Figure 2:**
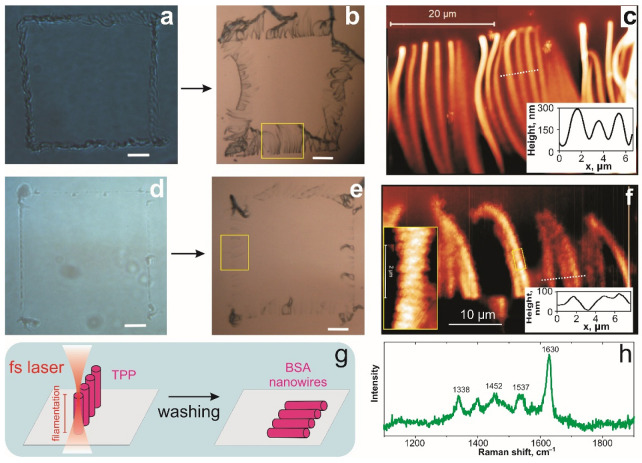
TPP of vertical BSA hydrogel nanowires: (**a**) Optical image of BSA hydrogel “walls” after polymerization under laser focused above the substrate; (**b**) optical image of “walls” after washing out non-polymerized BSA solution; (**c**) AFM image of “walls” marked by rectangular at (**b**). (**d**–**f**) The characterization of “walls” produced by laser focused close to substrate surface: optical image before (**d**) and after (**e**) washing; (**f**) AFM image of “walls” denoted by a rectangle of (**e**). The insert images on right (**c**, **f**): cross-section of individual nanowires. Left bottom insert of (**f**): enlarged AFM image of an individual nanowire with visible periodic sub-wavelength nanostructures; (**g**) scheme of BSA hydrogel nanowires fabrication by femtosecond pulsed laser; (**h**) Raman spectra of BSA hydrogel nanowires.

Both BSA and BSA@GO solutions were stored in a refrigerator at +4 °C and were stable within two weeks. The solutions were polymerized under UV light (5 W/cm^2^ at 254 nm) to prove the formation of polymer structures. To perform TPP, we first attached the 5 mm in diameter polydimethylsiloxane (PDMS) well to the glass cover slip. Following which, 30 µL of solution was added to the well and placed under the objective of the optical microscope to supply femtosecond laser pulses with 25 mW power and scanning speed of 1 µm/s. Note that there was not movement of the laser focused in Z axis during performing of TPP, and its variation is due to the non-planar substrate surface. We found that depending on the focus position of the laser that the nanowires of different diameters were formed (Figure 2). When laser focus is above the substrate, 3D polymerized “walls” grow perpendicular to the substrate surface (Figure 2a). The sample was washed, resulting in breaking down the “wall” and it parts falling onto a substrate (Figure 2b). We found that the “wall” consists of well-separated 30 ± 2 µm long nanowires with diameter of 320 ± 70 nm (Figure 2c). The soft nature of hydrogel results in variations of measured diameter of nanowires. Nevertheless, the diameters of nanowires are well below the femtosecond laser wavelength as a result providing sub-wavelength lithography. When the laser focus is close to the substrate surface, the length and diameter of BSA hydrogel nanowires can be decreased to 20 ± 2 µm and 70 ± 20 nm, respectively (Figure 2d–f).

The observed formation of TPP hydrogel nanowires can be explained in terms of the self-focusing effect of the laser beam due to the difference in liquid and polymerized hydrogel refraction indexes [21,22]. The effect provides an almost uniform cross-section of two-photon absorption over the whole filament length leading to high-aspect-ratio of the hydrogel nanowire (length/diameter ≈ 100) as shown in Figure 2c. Formation of sub-wavelength nanostructures with period of 300 ± 20 nm is well visible on the sidewalls of narrow nanowires (Figure 2f, insert). We assume that the effect of self-trapped beams plays a major role in the formation of nanowires in BSA and MB solution, generating “walls” perpendicular to the substrate (Figure 2g). When a femtosecond laser beam is focused close to the glass surface, self-focusing leads to the activation and directional moving of PI, causing nanowire polymerization. For a smaller diameter, it is possible to resolve the PI waves along the laser beam with a period of λ/2 leading to the periodical nanostructuring of the nanowire surface. After washing we observe the falling of “walls” resulting in directional nanowire positioning. The different positions of the fallen nanowires point out that these are individual isolated structures. The main bands in the Raman spectra (Figure 2h) of albumin were clearly observed [23]: 1338 cm^−1^ (deformation mode of CH bonds), 1452 cm^−1^ (deformation mode of CH_2_ bonds), and 1630 cm^−1^ (Amide I). The secondary structure of BSA when denaturized under ultrafast laser pulses changes its conformation causing Raman peaks shift [24]. An additional band of 1537 cm^−1^ responsible for C=C stretching and shift in main peaks appeared due to protein photochemical polymerization. 

**Figure 3:**
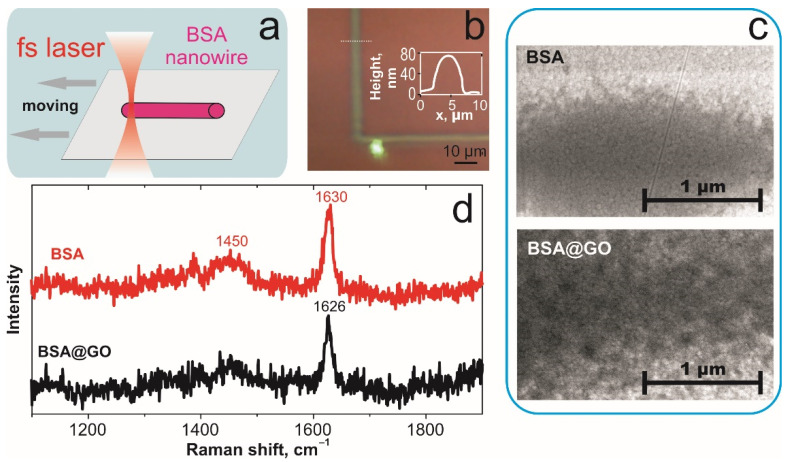
TPP of horizontal BSA hydrogel nanowires: (**a**) Scheme of DLW of BSA hydrogel nanowires on substrates; (**b**) optical image DLW BSA nanowires on silicon substrate. The insert image: cross section of DLW nanowire; (**c**) SEM image of BSA and BSA@GO hydrogel’s structure; (**d**) Raman spectra of BSA and BSA@GO hydrogel nanowires.

Some tasks of bio-inspired applications need precise and local positioning of individual nanowires. The direct laser writing (DLW) of hydrogels can be applied when moving substrate during the TPP process [25]. We performed DLW on both BSA and BSA@GO hydrogel nanowires on Si/SiO_2_ substrate (Figure 3a). The TPP structures had become wider by up to 5 µm with height of 100 ± 20 nm (Figure 3b). SEM imaging reveals the low concentration of GO flakes that are randomly distributed in the polymerized matrix while pristine BSA have uniform structure (Figure 3c). The GO concentration is too low to be resolved by Raman spectroscopy in hydrogel. The shift in Raman spectra for Amide I band to 1626 cm^−1^ in BSA@GO hydrogel (Figure 3d) can be due to direct interaction of BSA and GO flakes during femtosecond laser processing [17] or stronger interaction of water molecules with defects in GO [26]. The interaction of albumin with GO leads to its partial unfolding that can be modulated by pH and ionic strength of solution [20,27]. It should be noted that during TPP there is possibility for reduction of GO resulting in increase of active oxygen groups [28,29]. In turn, it can stimulate the BSA polymerization close to graphene flakes. After the photochemical reduction of GO, the number of negative charges is decreased on the surfaces, which should decrease the repulsive energy of BSA [20] and hence increase the BSA on GO surface coverage.

**Figure 4:**
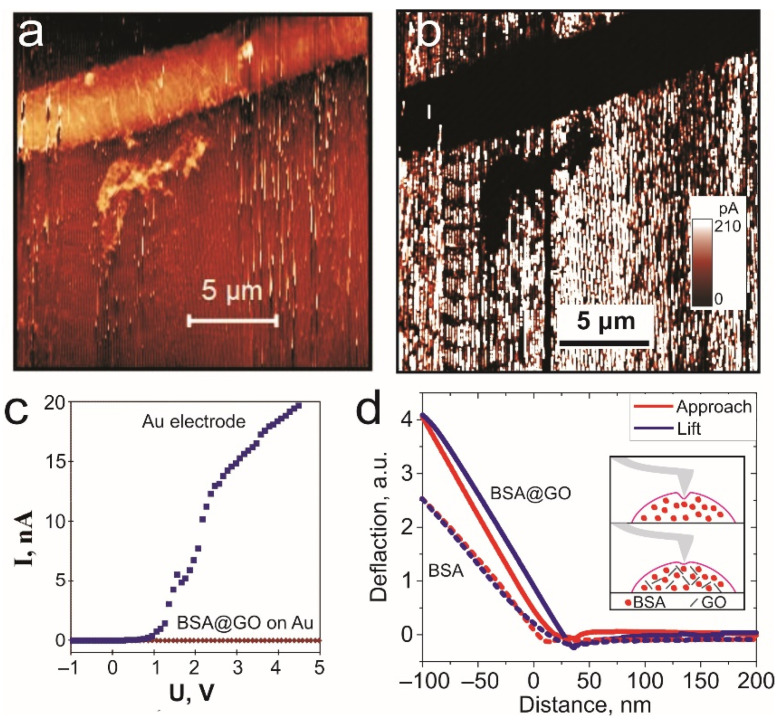
Local characterization of BSA@GO TPP nanowires: (**a**) AFM image of BSA@GO nanowire on gold substrate; (**b**) 2D map of spreading current of BSA@GO nanowire on gold. (**c**) Current vs. voltage spectra of contact between conductive AFM probe and BSA@GO nanowire and Au surface. (**d**) Deflection–distance curves of the BSA and BSA@GO nanowires. In the insert: schematics of the indentation of BSA and BSA@GO materials.

The local electrical and mechanical properties of BSA and BSA@GO nanowires were investigated using atomic force microscopy with conductive cantilevers (for electrical measurement) and cantilevers with higher force constant (for nanoindentation characterization). For the former task we polymerized nanowires by DLW on gold substrate (Figure 4a,b). We observed good insulation properties of BSA@GO nanowires due to low concentration of GO in initial solution that prevents the formation of a percolating graphene network inside the BSA matrix. We discovered that there is an energy gap even between cantilever and gold surface contact. BSA molecules provide excellent properties of surfactant tending to form monolayer films on any hydrophobic surface (called “corona”) [20]. Thus, the monolayer of non-polymerized BSA molecules on the gold surface can cause non-Ohmic contact even on flat gold surfaces.

Nanoscale mechanical properties of BSA@GO nanowires were investigated by local probing using standard silicon cantilevers. Effective Young’s modulus of graphene oxide was estimated as ~200 GPA [30]. The addition of a small portion of GO to the BSA composite can greatly increase BSA@GO hardness. The cantilever deflection curves were used to estimate deflection depth and elastic properties (Table 1). The hardness is proportional to the maximal load on a deflection curve [31] and we can estimate the increase of hardness for BSA@GO compared to BSA from Figure 4d. The indentation depth decreases when the GO is added to the BSA hydrogel. We assume that the improvement of mechanical properties of BSA@GO hydrogel nanowires was due to rearrangement and structuring of BSA molecules during the polymerization close to the GO surface. 

Increase in concentration of GO can both gradually improve the electrical properties and effect on mechanical properties of composite nanowires. Nevertheless, we have demonstrated that the gradual change in composite properties starts even for small concentrations of GO. Previously we demonstrated the biocompatibility of nanocomposites of BSA [14]. Nevertheless, additional investigation on cell proliferation has to be done for proper analysis of BSA@GO biocompatibility.

## 4. Conclusions

We have suggested the BSA and BSA@GO nanowires formation based on two-photon polymerization of protein photoactive solution. The novel route for nanofilaments growth with diameter down to 100 nm and aspect ratio up to 100 was demonstrated. The local electrical and mechanical properties of individual BSA@GO nanowires were investigated. The nanowires still provide good isolating properties for 0.005% GO concentration. Mechanical properties of nanowires were greatly improved for the BSA@GO composite. The hardness was increased up to 1.6 times. This suggested route provides novel bioinspired technology for functional biomimicking materials based on photoactive protein solutions and soluble graphene oxide.

## Figures and Tables

**Table 1:** The local mechanical properties of BSA and BSA@GO nanowires.

Mechanical Parameters	BSA	BSA@GO
Increase of hardness	1	1.6
Indentation depth	35 nm	20 nm

## Data Availability

Not applicable.
